# Computer Optimization of Biodegradable Nanoparticles Fabricated by Dispersion Polymerization

**DOI:** 10.3390/ijerph13010047

**Published:** 2015-12-22

**Authors:** Emmanuel O. Akala, Simeon Adesina, Oluwaseun Ogunwuyi

**Affiliations:** Center for Drug Research and Development, Department of Pharmaceutical Sciences, College of Pharmacy, Howard University, 2300 4th Street NW, Washington, DC 20059, USA; simeon.adesina@howard.edu (S.A.); temytayoo@yahoo.com (O.O.)

**Keywords:** nanoparticles, quality by design (QbD), d-optimal mixture design, statistical design of experiments, computer optimization, dispersion polymerization

## Abstract

Quality by design (QbD) in the pharmaceutical industry involves designing and developing drug formulations and manufacturing processes which ensure predefined drug product specifications. QbD helps to understand how process and formulation variables affect product characteristics and subsequent optimization of these variables *vis-à-vis* final specifications. Statistical design of experiments (DoE) identifies important parameters in a pharmaceutical dosage form design followed by optimizing the parameters with respect to certain specifications. DoE establishes in mathematical form the relationships between critical process parameters together with critical material attributes and critical quality attributes. We focused on the fabrication of biodegradable nanoparticles by dispersion polymerization. Aided by a statistical software, d-optimal mixture design was used to vary the components (crosslinker, initiator, stabilizer, and macromonomers) to obtain twenty nanoparticle formulations (PLLA-based nanoparticles) and thirty formulations (poly-ɛ-caprolactone-based nanoparticles). Scheffe polynomial models were generated to predict particle size (nm), zeta potential, and yield (%) as functions of the composition of the formulations. Simultaneous optimizations were carried out on the response variables. Solutions were returned from simultaneous optimization of the response variables for component combinations to (1) minimize nanoparticle size; (2) maximize the surface negative zeta potential; and (3) maximize percent yield to make the nanoparticle fabrication an economic proposition.

## 1. Introduction

One of the great challenges in drug development and medicine today is finding more effective forms of treatment for a large number of life-threatening but curable diseases, such as cancer. At the moment, there is an imbalance between the knowledge of cancer biology and the success achieved in cancer treatment: efforts in the treatment of cancer have not met with much success [1,2]. To achieve the goal of eliminating death and suffering from cancer, the USA National Cancer Institute has embraced the power of nanotechnology to radically change the procedure for diagnosis, imaging and treating cancer. The integration of the developments in nanotechnology with advances in cancer research can be done using polymeric nanoparticles which are capable of specific delivery of large amounts of single or multiple therapeutic agents as well as imaging agents embedded in the core per targeting biorecognition event compared to simple immunotargeted drugs. Both targeting (spatial/distribution control) and controlled release (temporal control) of therapeutic agents can be achieved [3,4].

The evolution of nanoparticles for biomedical applications has moved from the first generation nanoparticles (mainly suitable for liver targeting) through the second generation (stealth nanoparticles for long blood circulation and passive targeting) to the third generation nanoparticles with molecular recognition [1,5]. The fourth generation has been dubbed theranostics: multifunctional nanoscale devices which allow for a combination of diagnostic agent with a therapeutic agent and even a reporter of therapeutic efficacy in the same nanodevice package [6]. Aside from biocompatibility and biodegradability, the physicochemical properties of nanoparticles (size and surface modification, among others) and their interactions with biological systems are important considerations in their design and fabrication. Polymeric nanoparticles can be prepared mainly by two methods: (i) dispersion of preformed polymers and (ii) polymerization of monomers (*i.e.*, *in situ* polymerization). *In situ* polymerization of monomers and crosslinkers offers many advantages including one-pot synthesis of nanoparticles [7]. Our laboratory has focused on surfactant free, free radical dispersion polymerization (*in situ* polymerization) technique for the fabrication of stealth crosslinked nanoaprticles for biomedical applications [2,7,8,9,10]. We report here our efforts on the development of stealth biodegradable cross-linked nanoparticles by dispersion polymerization suitable for the delivery of bioactive agents.

The relatively new concept of quality by design (QbD) and process analytical technology (PAT) in pharmaceutical dosage form design and development, already incorporated into automakers’ production principles, involves designing and developing drug formulations and manufacturing processes which ensure predefined drug product specifications. It is believed that product and process understanding is a key element of QbD-PAT [7,10,11]. Thus an important part of QbD-PAT is to understand how process and formulation variables affect product characteristics and subsequent optimization of these variables *vis-à-vis* the final specifications. Statistical design of experiments (DoE) is a well-established method for identifying important parameters in pharmaceutical dosage form design and for optimizing the parameters with respect to certain specifications [7,10,11,12]. Two major approaches to the design of experiments to be able to examine all of the variables simultaneously are factorial and mixture experimental designs [7,10]. The statistical experimental designs involving mixture methodology is an efficient method for studying products made from components at various levels. We used d-optimal mixture design for experimental design, analysis and optimization. When a formulation is a mixture of various components (proportion of the constituents) as studied in our work and the levels of the components are constrained, d-optimal mixture design is more useful than a factorial design because it accounts for the dependence of response on proportionality of constituents.

## 2. Materials and Methods

Two types of nanoparticles were fabricated and characterized as discussed previously: poly-l-lactide-based [10] and poly-ɛ-caprolactone-based nanoparticles [7].

**Figure 1:**
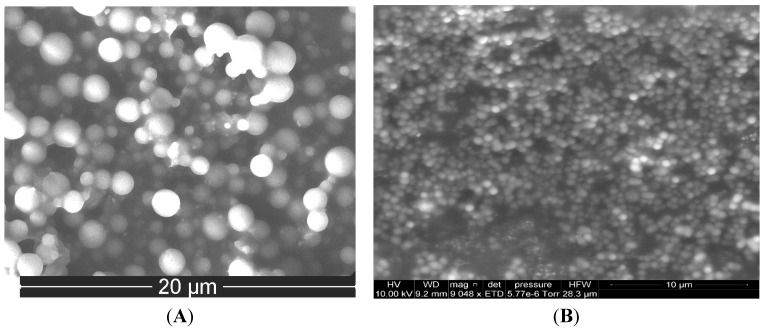
Typical SEM images of blank nanoparticles prepared by *in situ* dispersion polymerization (**A**) Poly-l-lactide-based nanoparticles; (**B**) Poly-ɛ-caprolactone-based nanoparticles.

They were characterized for surface morphology (scanning electron microscopy (SEM)), particle size (dynamic light scattering (DLS) using Zetasizer Nano-ZS (Malvern Instruments, Malvern, UK), yield, and surface zeta potential (Zetasizer Nano-ZS). Typical electron micrographs are shown in Figure 1.

We used mixture design (d-optimal mixture statistical experimental design) in this work for the response surface method (RSM). The responses (particle size and percent yield (for poly-l-lactide-based nanoparticles) and particles size and surface zeta potential (for poly-ɛ-caprolactone-based nanoparticles) are functions of the proportions of the formulation variables investigated: macromer, initiator, stabilizer, and crosslinker. Based on preliminary data, constraints were introduced to the proportions of the components to allow the fabrication of smooth spherical particles. In d-optimal mixture design, there are restrictions on component proportions such that a lower and upper limits are specified [7,10,13,14,15,16]. Aided by statistical software for the design of the experiments and analysis of the data (Design-Expert^®^, Stat-Ease Inc., Minneapolis, MN, USA), and using d-optimal mixture statistical experimental design, we varied the components (critical material attributes (CMAs): crosslinker, initiator, stabilizer and poly-l-lactide-HEMA macromonomer) to obtain twenty nanoparticle formulations for poly l-lactide-based nanoparticles (Table 1) and thirty nanoparticle formulations (Table 2) for poly-ɛ caprolactone-based nanoparticles. The particle size for poly-l-lactide-based nanoparticles ranges from 261 to 326 nm; while the polydispersity index (PDI) ranges from 0.20 to 0.29. For poly-ɛ-lcaprolactone based nanoparticles, the particle size obtained ranges from 130 nm to 788 nm; while PDI ranges from 0.133 to 0.605. The particle size distribution is given by the PDI. A PDI value of <0.1 indicates a homogenous monodisperse formulation; while a PDI of >0.3 indicates polydispersity with variations in particle size.

**Table 1:** Composition and response of d-optimal mixture experiemntal design for the fabrication of stealth poly-l-lactide-based nanoparticles.

Standard Order	Run Order	A: Crosslinking Agent (mmol)	B: Initiator System (mmol)	C: Stabilizer (PEG-MMA) (mmol)	D: Macromonomer (mmol)	Response 1 (Particle Size: nm)	Response 2 (Percent Yield %)
9	1	0.048	0.359	0.304	0.289	297.6	56.87
8	2	0.056	0.285	0.215	0.445	306.6	28.99
1	3	0.087	0.377	0.091	0.445	261.5	62.39
5	4	0.018	0.565	0.214	0.204	270.6	35.26
19	5	0.087	0.377	0.091	0.445	268.1	58.82
12	6	0.042	0.452	0.259	0.247	286.4	31.88
20	7	0.018	0.565	0.214	0.204	295.8	61.08
17	8	0.087	0.183	0.304	0.426	326.4	31.13
14	9	0.018	0.312	0.304	0.367	290.3	41.19
15	10	0.053	0.415	0.148	0.384	322.3	45.15
3	11	0.055	0.625	0.115	0.204	293.1	47.87
4	12	0.087	0.183	0.304	0.426	322.9	34.18
13	13	0.036	0.522	0.163	0.279	320.7	43.08
2	14	0.018	0.446	0.091	0.445	313.1	24.43
11	15	0.087	0.500	0.091	0.321	273.3	43.32
16	16	0.018	0.446	0.091	0.445	293.8	35.08
18	17	0.055	0.625	0.115	0.204	305.9	39.22
6	18	0.018	0.234	0.304	0.445	320.9	35.98
7	19	0.018	0.625	0.091	0.265	295.6	44.71
10	20	0.087	0.394	0.198	0.321	314.9	37.59

**Table 2:** Composition and response of d-optimal mixture experiemntal design for the fabrication of stealth poly-ɛ-caprolactone-based nanoparticles.

Standard Order	Run Order	A: Crosslinking Agent (mmol)	B: Initiator System (mmol)	C: Stabilizer (PEG-MMA) (mmol)	D: Macromonomer (mmol)	Response 1 (Particle Size: nm)	Response 2 (Negative Zeta Potential: mV)
9	1	0.018	0.358	0.422	0.203	691.5	14.1
11	2	0.038	0.298	0.449	0.215	131.4	36
20	3	0.021	0.480	0.255	0.244	675.9	20.5
19	4	0.027	0.332	0.401	0.240	378.3	31.1
25	5	0.027	0.411	0.311	0.251	328.3	15.3
2	6	0.016	0.499	0.305	0.180	653	29.7
6	7	0.033	0.379	0.397	0.191	148.4	31.7
17	8	0.034	0.502	0.237	0.227	228	28.2
5	9	0.024	0.512	0.279	0.185	235.6	27.4
7	10	0.034	0.438	0.334	0.194	130.8	22
18	11	0.034	0.502	0.237	0.227	224	27.7
14	12	0.019	0.475	0.287	0.219	749	23.1
21	13	0.038	0.407	0.308	0.247	181.3	15.5
27	14	0.045	0.356	0.342	0.257	130.8	36.6
29	15	0.023	0.364	0.350	0.263	687	20
10	16	0.018	0.566	0.213	0.204	635.5	21.4
28	17	0.022	0.451	0.269	0.258	603.6	29.2
23	18	0.032	0.345	0.373	0.249	255.1	25
8	19	0.035	0.556	0.209	0.201	131.6	0.05
30	20	0.037	0.389	0.293	0.281	241	0.04
24	21	0.027	0.411	0.311	0.251	372.3	17.4
1	22	0.024	0.462	0.347	0.167	242.7	32.4
26	23	0.044	0.442	0.262	0.252	152.6	−0.11
13	24	0.019	0.475	0.287	0.219	550.5	0.07
4	25	0.024	0.512	0.279	0.185	224	35.8
12	26	0.024	0.366	0.392	0.218	390.7	33.9
16	27	0.020	0.388	0.368	0.224	703	0.04
22	28	0.038	0.407	0.308	0.247	263.1	0.04
15	29	0.019	0.304	0.457	0.220	788.6	0.01
3	30	0.021	0.410	0.384	0.185	325.1	−0.08

## 3. Results

### 3.1. Data Analysis, Generation of Scheffe Polynomials from Data Analysis

#### Followed by Optimization

The selection of the best models for modeling the response variables (particle size and yield for poly-l-lactide-based nanoparticles) and particles size and surface zeta potential for poly-ɛ-caprolactone-based nanoparticles is important since the fitted models will be used to predict the variables following simultaneous numerical optimization [17]. With a mixture design, the response determined by any possible component mixtures can be identified by a point in the experimental domain called the design space. When working with three different variables (components), the experimental domain corresponds to an equilateral triangle with the vertices corresponding to the pure components while different points within the design space correspond to a mixture of components [7,10,18]. In this work, four components (macromonomer, initiator system, crosslinking agent and stabilizer) were combined to prepare nanoparticles; however, the proportion of the macromonomer was kept constant in all the experiments thereby yielding a triangular experimental domain. Further, as a result of constraints introduced, the region of interest (design space) that allows for the formation of smooth spherical particles is only a fraction of the possible experimental domain.

### 3.2. Poly-l-Lactide-Based Nanoparticles

Model fitting to the data (Table 1) was carried out and the quadratic model was found significant and was selected. To improve the model, insignificant terms were removed by backward elimination. Analysis of variance (ANOVA) of the selected model and terms (Table 3) reveal that the selected model is significant (*p* = 0.0020). Further, the model (Scheffe Polynomial) was also selected based on the estimation of several statistical parameters: multiple correlation coefficient (*R*^2^), adjusted multiple correlation coefficient (adjusted *R*^2^) and the predicted residual sum of squares (PRESS). Also, “lack of fit” was not statistically significant (*p* = 0.4994) which is desirable. The resulting model (Scheffe polynomial) is shown in Equation (1) below:

Particle size (nm) = −1772.43 (A) + 275.78 (B) − 1089.88 (C) + 270.51 (D) + 2481.55 (AB) + 7504.21 (AC) + 1453.29 (BC) + 2509.21 (CD)
(1)

where: A = Crosslinker (mmol); B = Initiators (mmol); C = Stabilizer (mmol); D = Macromonomer (mmol).

**Table 3:** Analysis of variance table for particle size (Poly-l-lactide-based nanoparticles).

Source	Sum of Squares	df	Mean Square	F-Value	*p*-Value	
Model	6011.26	7	858.75	6.83	0.0020	s
Linear mixture	1431.12	3	477.04	3.79	0.0401	s
AB	693.79	1	693.79	5.52	0.0368	s
AC	2723.37	1	2723.37	21.65	0.0006	s
BC	694.88	1	694.88	5.52	0.0367	s
CD	1247.85	1	1247.85	9.92	0.0084	s
Residual	1509.25	12	125.77			
Lack of Fit	895.66	7	127.95	1.04	0.4994	ns
Pure Error	613.59	5	122.72			
Cor Total	7520.52	19				

Notes: s = significant; ns = not significant

**Figure 2:**
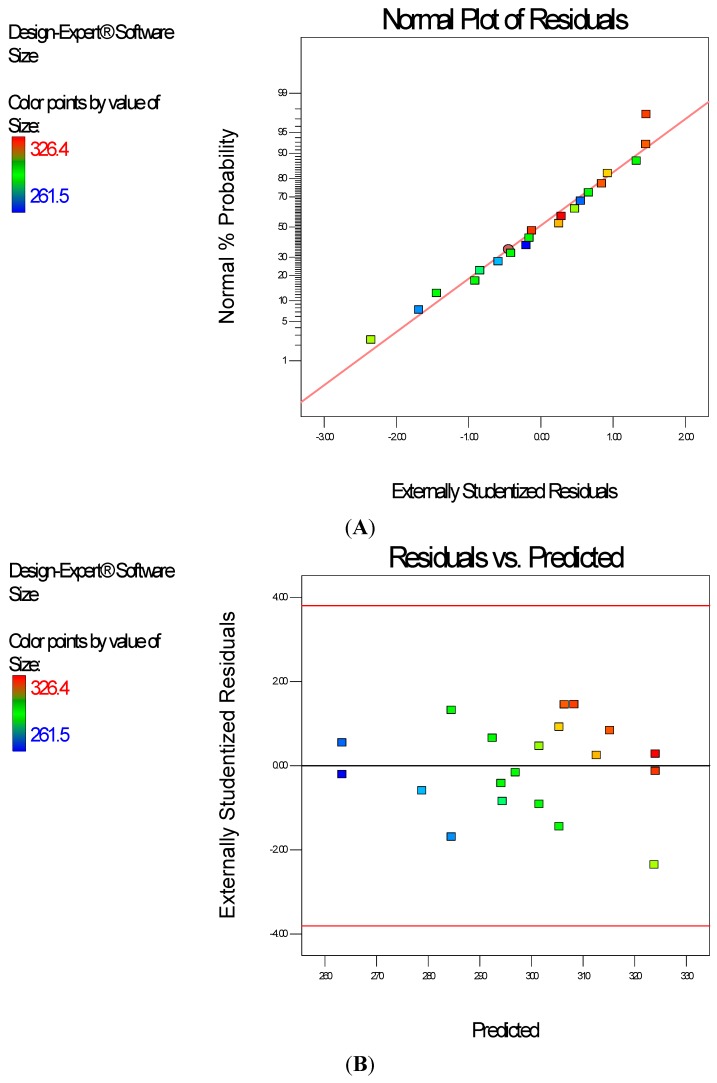
Diagnostic plots the particle size data of polylactide-based nanoparticles: (**A**) Normal Plot of Residuals; (**B**) Residuals *vs.* Predicted.

**Figure 3:**
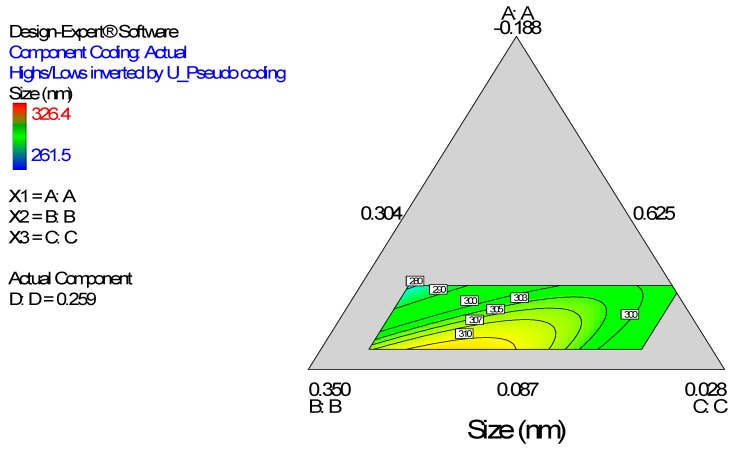
Model graph showing the design space and variation in particle size as a function of the mixture composition. A = Crosslinking agent; B = Initiators; C = Stabilizer and D = Macromonomer (Polylactide-based nanoparticles).

Diagnostic plots (Figure 2) show the validity of the model. The normal probability plot of the residuals (Figure 2A) is the most important diagnostic and it checks for non-normality in the error term. A linear normal probability plot of the residuals, which indicates normality in the error term, was obtained. Figure 2B shows a diagnostic plot that tests the assumption of constant variance. Both plots show no problem with our data. The Scheffe polynomial (Equation (1)) was used to generate the model graph (Figure 3) which shows the design space and variation in particle size as a function of the mixture composition. (A = Crosslinking agent; B = Initiators; C = Stabilizer and D = Macromonomer) (Poly-l-lactide-based nanoparticles). The predicted sizes for the four solutions are 276.7 nm, 283.6 nm, 291.6 nm and 302.7 nm while the experimentally obtained sizes are 244.2 nm ± 4.20 nm, 246.6 nm ± 0.87 nm, 250.4 nm ± 4.04 nm and 271 nm ± 4.62 nm respectively (Table 4). The corresponding polydisperity index (PDI) values are 0.23, 0.28, 0.27 and 0.29, respectively.

**Table 4:** Analysis of variance table for percent yield (Poly-l-lactide-based nanoparticles).

Source	Sum of Squares	df	Mean Square	F-Value	*p*-Value (Prob > F)	
Model	1163.07	5	232.61	2.98	0.0488	s
*Linear Mixture*	*326.63*	*3*	*108.88*	*1.40*	*0.2854*	*ns*
*AB*	*508.70*	*1*	*508.70*	*6.52*	*0.0230*	*s*
*AD*	*822.41*	*1*	*822.41*	*10.54*	*0.0059*	*s*
Residual	1092.30	14	78.02			
*Lack of Fit*	*653.82*	*9*	*72.65*	*0.83*	*0.6211*	*ns*
*Pure Error*	*438.48*	*5*	*87.70*			
Cor Total	2255.38	19				

Notes: s = significant; ns = not significant.

A similar model fitting was done for percent yield as shown in Table 4. The resulting model (Scheffe polynomial) is shown in Equation (2) below:

Percent Yield = −2011.036 (A) + 60.513 (B) + 126.177 (C) − 46.488 (D) + 1935.312 (AB) + 3996.599 (AD)
(2)

where: A = Crosslinker (mmol); B = Initiators (mmol); C = Stabilizer (mmol); D = Macromonomer (mmol)

**Figure 4:**
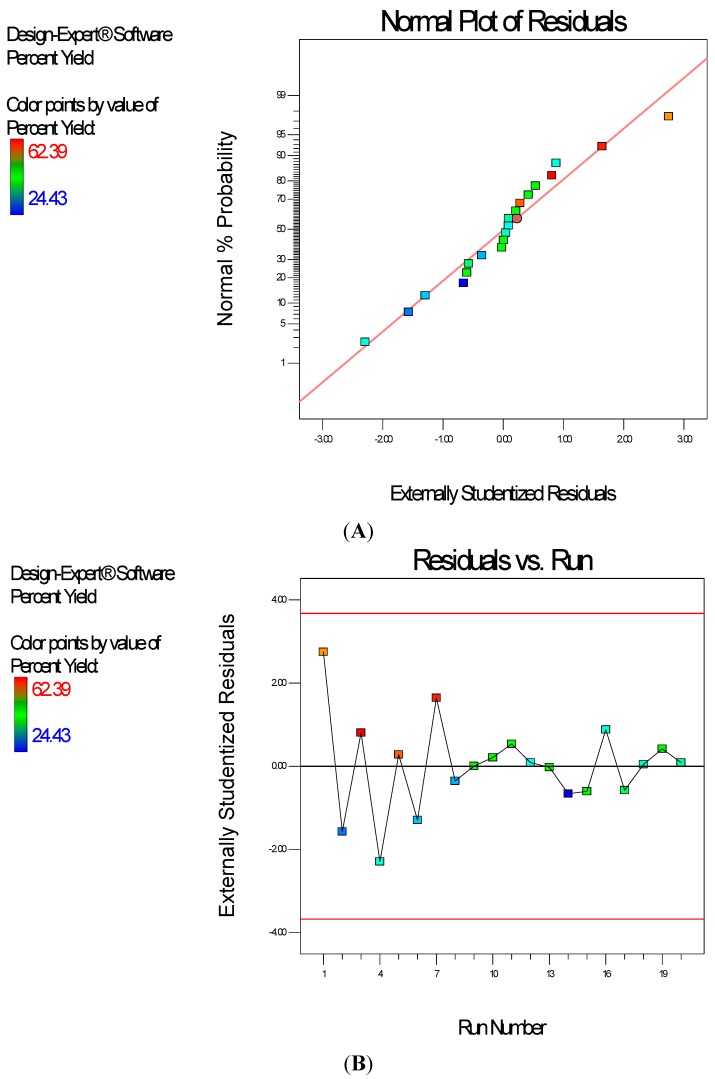
Diagnostic plots the percent yield data of Polylactide-based nanoparticles: (**A**) Normal Plot of Residuals; (**B**) Residuals *vs.* Run.

**Figure 5:**
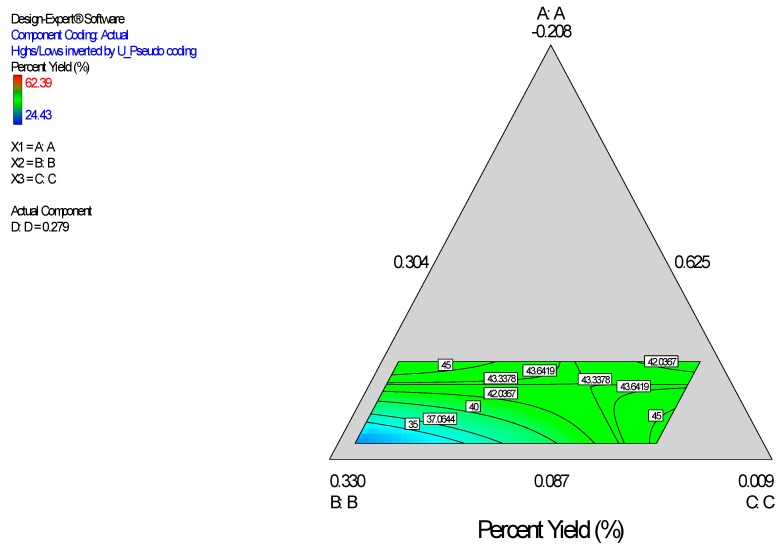
Model graph showing the design space and variation in percent yield as a function of the mixture composition. A = Crosslinking agent; B = Initiators; C = Stabilizer and D = Macromonomer (Polylactide-based nanoparticles).

Diagnostic plots (Figure 4) show the validity of the model. The Scheffe polynomial (Equation (2)) was used to generate the model graph (Figure 5), which shows the design space and variation in percent yield as a function of the mixture composition. A = Crosslinking agent; B = Initiators; C = Stabilizer and D = Macromonomer (Polylactide-based nanoparticles).

### 3.3. Poly-ɛ-Caprolactone-Based Nanoparticles

Logarithmic transformation was carried out before model fitting to particle size data (Table 2). The quadratic model was found significant and was selected. To improve the model, insignificant terms were removed by backward elimination. Analysis of variance (ANOVA) of the selected model and terms (Table 5) reveals that the selected model is significant (*p* < 0.0001). The linear mixture (component linear terms) and the square of A term (crosslinker term) are significant: *p* < 0.0001 and *p* = 0.0005 respectively. Additionally, “lack of fit” is not significant (*p* = 0.6921). Non-significant lack of fit is good as our desire is for the model to fit. The “Pred R-Squared” of 0.9326 is in reasonable agreement with the “Adj R-Squared” of 0.9455. Adequate Precision measures the signal to noise ratio. A ratio greater than 4 is desirable. The ratio of 27.332 obtained in this work indicates an adequate signal. Consequently, this model can be used to navigate the design space.

**Table 5:** Analysis of variance table for particle size (Poly-ɛ-caprolactone-based nanoparticles.

Source	Sum of Squares	df	Mean Square	F-Value	*p*-Value (Prob > F)	
Model	1.98	4	0.49	126.80	<0.0001	s
*Linear Mixture*	*1.92*	*3*	*0.64*	*163.82*	*<0.0001*	s
*A^2^*	*0.061*	*1*	*0.061*	*15.71*	*0.0005*	s
Residual	0.098	25	3.904e-003			
*Lack of Fit*	*0.074*	*20*	*3.691e-003*	*0.78*	*0.6921*	*ns*
*Pure Error*	*0.024*	*5*	*4.756e-003*			
Cor Total	2.08	29				

Notes: s = significant; ns = not significant.

The empirical model (Scheffe polynomial) is shown in Equation (3) below:

Log_10_ (Size) = −74.47882 (A) + 3.04138 (B) + 3.14184 (C) + 7.33688 (D) + 744.06483 (A^2^) (3)

where: A = Crosslinker (mmol); B = Initiators (mmol); C = Stabilizer (mmol); D = Macromonomer (mmol).

**Figure 6:**
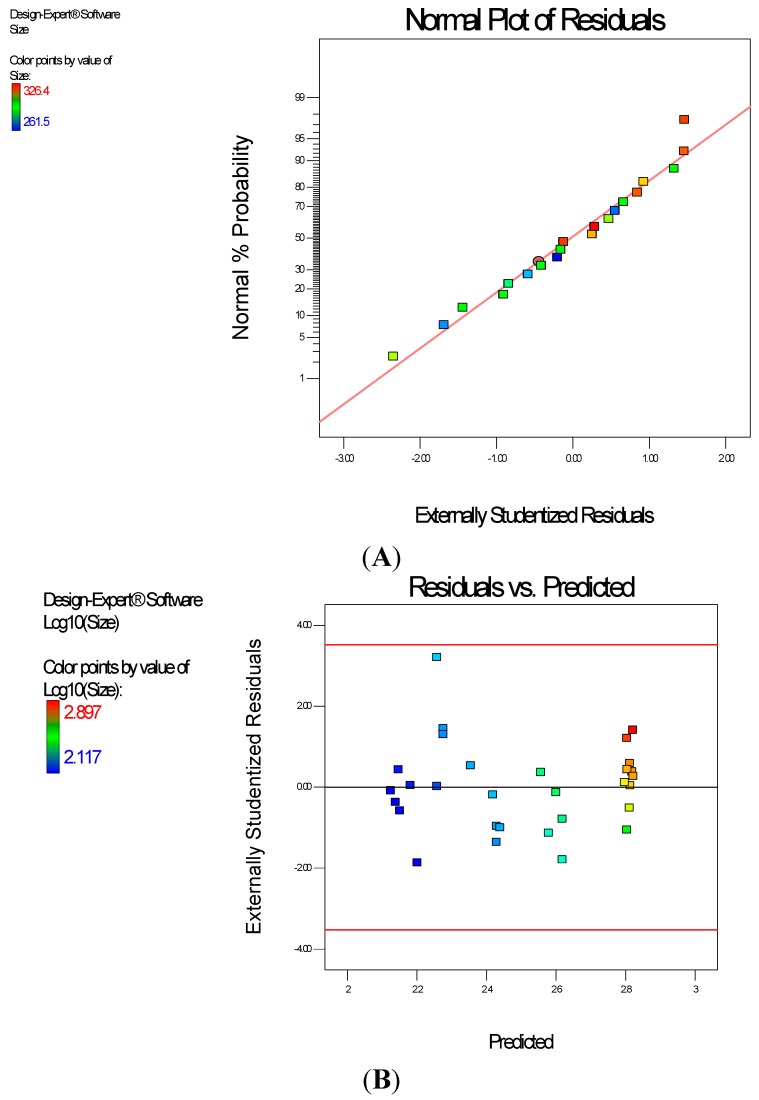
Diagnostic plots the particle size data of poly-ɛ-aprolactone-based nanoparticles: (**A**) Normal Plot of Residuals (**B**) Residuals *vs.* Predicted.

**Figure 7:**
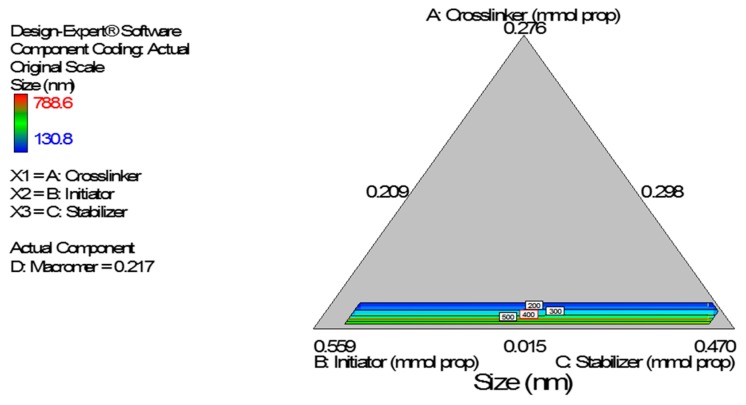
Model graph showing the design space and variation in particle size as a function of the mixture composition. A = Crosslinking agent; B = Initiators; C = Stabilizer and D = Macromonomer (poly-ɛ-caprolactone-based nanoparticles).

Diagnostic plots show the validity of the model (Figure 6). The normal probability plot of the residuals is the most important diagnostic plot; it checks for non-normality in the error term. A linear normal probability plot of the residuals was obtained which indicates normality in the error term and therefore there is no problem with our data. Further, Residuals *vs.* Predicted tests the assumption of constant variance and it should be a random scatter within the upper and lower boundaries. The Scheffe polynomial (Equation (3)) was used to generate the model graph (Figure 7) which shows the design space and variation in particle size as a function of the mixture composition. A = Crosslinking agent; B = Initiators; C = Stabilizer and D = Macromonomer (Poly-ɛ-caprolactone based nanoparticles).

Following square root transformation, model fitting for zeta potential data was carried out. Quadratic model was found significant and the model was selected. To improve the model, insignificant terms were removed by backward elimination. Analysis of variance (ANOVA) (Table 6) reveals that the selected model is significant (*p* = 0.0437). The linear mixture terms (component linear terms) are not significant (*p* = 0.7487); the quadratic term of A (crosslinker) by C (stabilizer) is significant *p* = 0.0037. In addition, “lack of fit” is not significant (*p* = 0.4389). Non-significant lack of fit is good; we want the model to fit. Adequate precision measures the signal to noise ratio. The ratio of 6.96 indicates an adequate signal. This model can be used to navigate the design space.

**Table 6:** Analysis of variance table for zeta potential (Poly-ɛ-caprolactone-based nanoparticles.

Source	Sum of Squares	Df	Mean Square	F-Value	*p*-Value (Prob > F)	
Model	43.01	4	10.75	2.87	0.0437	s
L*inear Mixture*	*4.58*	3	*1.53*	*0.41*	*0.7487*	*ns*
*AC*	*38.43*	*1*	*38.43*	*10.27*	*0.0037*	s
Residual	93.56	25	3.74			
*Lack of Fit*	*77.93*	*20*	*3.90*	*1.25*	*0.4389*	*ns*
*Pure Error*	*15.63*	*5*	3.13			
Cor Total	136.57	29				

Notes: s = significant; ns = not significant

The empirical model (Scheffe polynomial) is shown in Equation (4) below:

Sqrt (Negative Surface Zeta Potential + 0.20) = −644.92273 (A) + 24.45442 (B) − 32.18851 (C) + 12.26659 (D) + 2130.94151 (AC)
(4)

where: A = Crosslinker (mmol); B = Initiators (mmol); C = Stabilizer (mmol); D = Macromonomer (mmol).

**Figure 8:**
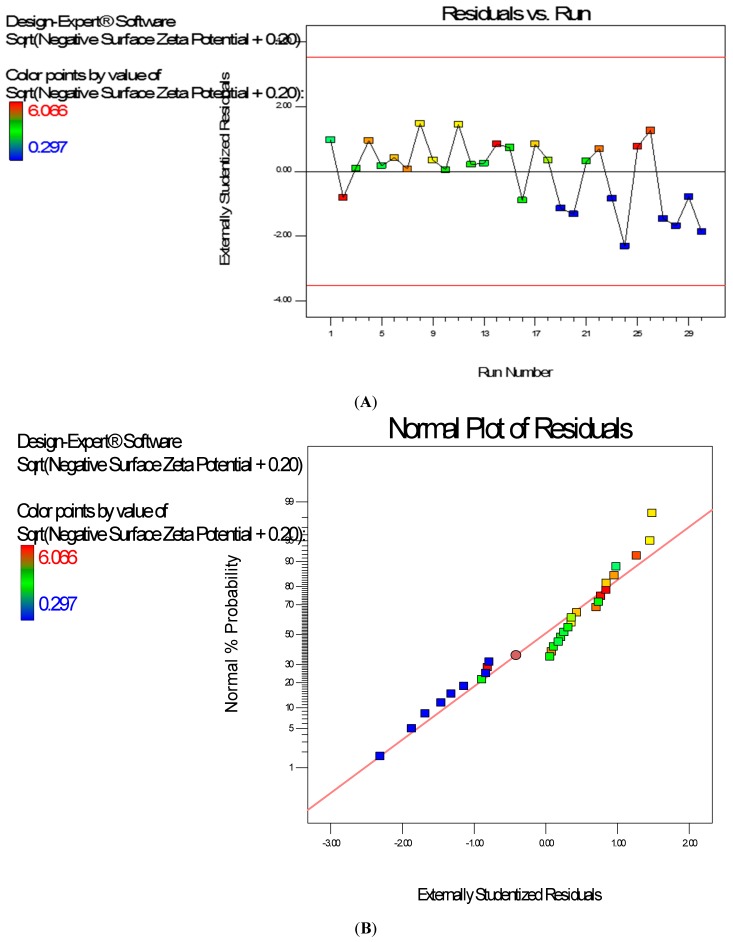
Typical Diagnostic plots the zeta potential data of poly-ɛ-caprolactone-based nanoparticles (**A**) Residuals *vs.* Experimental Run (should show a random scatter); (**B**) Normal Plot of Residuals (should give a straight line).

**Figure 9:**
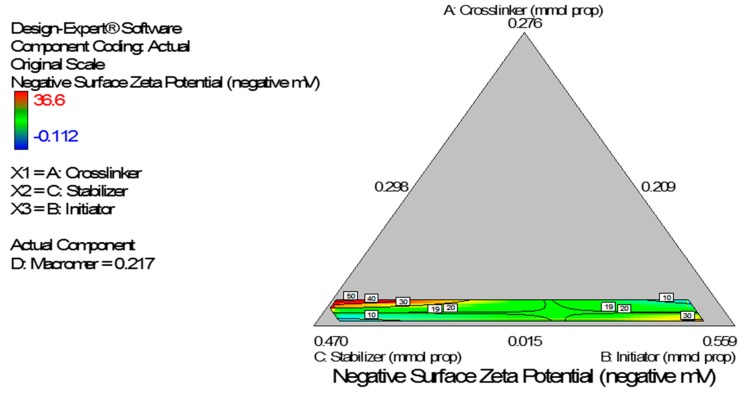
Model graph showing the design space and variation in negative zeta potential as a function of the mixture composition. A = Crosslinking agent; B = Initiators; C = Stabilizer and D = Macromonomer (poly-ɛ-caprolactone-based nanoparticles).

Diagnostic plots show the validity of the model (Figure 8). The Scheffe polynomial (Equation (4)) was used to generate the model graph (Figure 9) which shows the design space and variation in zeta potential as a function of the mixture composition. A = Crosslinking agent; B = Initiators; C = Stabilizer and D = Macromonomer (Poly-ɛ-caprolactone based nanoparticles).

## 4. Discussion

Scheffe polynomial models were generated to predict particle size (nm) and percent yield for poly-l-lactide-based nanoparticles as functions of the composition of the formulations. The models are shown in Equations (1) and (2). Further, Scheffe polynomial models were generated to predict particle size (nm) and zeta potential (mV) for poly-ɛ-caprolactone-based nanoparticles as functions of the composition of the formulations.

The models are shown in Equations (1) and (2).

### 4.1. Simultaneous Numerical and Graphical Optimizations of Nanoparticle Size and Percent Yield for Poly-l-Lactide-Based Nanoparticles

**Figure 10:**
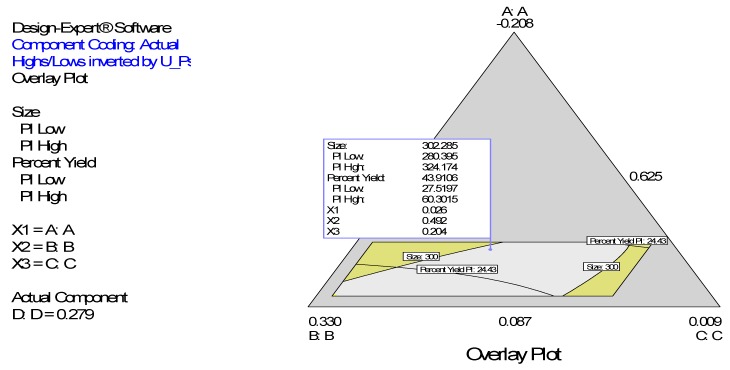
Simultaneous graphical optimization (overlay plot) of the design space variation in particle size and % yield as functions of the mixture composition. A = crosslinking agent; B = initiators; C = stabilizer and D = macromonomer.

Following simultaneous numerical optimization of nanoparticle size and percent yield of poly-l-lactide-based nanoparticles using Equations (1) and (2), four solutions were returned. Three of the solutions were used to fabricate nanoparticles to compare the predicted values with the actual laboratory values. The observations from the confirmation experiments are within the confirmation 95% prediction interval (95% PI low and 95% PI high), where PI is point prediction, showing the confirmation of the models. A typical overlay plot is shown in Figure 10. The focus on particle size and yield in this aspect of the work is based on the fact that particle size plays a key role in determining body distribution of nanoparticles after *in vivo* administration by injection and in facilitating their access to cancer cells (internalization) either by passive or active targeting to tumors [19,20]. Optimization of the nanoparticle fabrication for a high percent yield will make the drug development effort an economic proposition.

### 4.2. Simultaneous Numerical and Graphical Optimizations of Nanoparticle Size and Zeta Potential for Poly-Ɛ-Caprolactone-Based Nanoparticles

**Figure 11:**
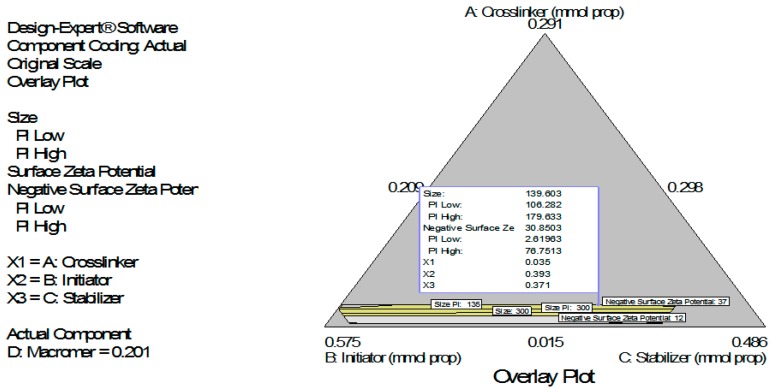
Simultaneous graphical optimization (overlay plot) of the design space variation in particle size and zeta potential as functions of the mixture composition. A = crosslinking agent; B = initiators; C = stabilizer and D = macromonomer.

Following simultaneous numerical optimization of nanoparticle size and nanoparticle surface zeta potential using the two models (Equations (3) and (4)), ten solutions were returned. Three of the solutions were used to fabricate nanoparticles to compare the predicted values with the actual laboratory values. The observations from the confirmation experiments are within the confirmation 95% prediction interval (95% PI low and 95% PI high), showing the confirmation of the models (Figure 11). As indicated earlier, we showed interest in particle size because particle size plays an important role in determining the drug release behavior of drug-loaded nanoparticles and the fate of the nanoparticles after *in vivo* administration [19,20]. The particles should be small enough to avoid the mechanical spleen or lung filtering effects. Moreover, the cells of the reticuloendothelial system (RES) or mononuclear phagocyte system recognize and rapidly clear nanoparticles from the circulation by phagocytosis and RES uptake has been shown to increase with particle size [21].

Zeta potential data in this work show predominantly negative values. Following injection into the blood stream, nanoparticles with a positive zeta potential pose a threat of causing transient embolism and rapid clearance compared to negatively charged particles [22]. Consequently, we decided to carry out simultaneous numerical optimization on particle size (with emphasis on minimization) and zeta potential (with emphasis on maximization of the negative zeta potential values).The overlay plots (Figure 10 and Figure 11) show the regions meeting the specifications for the optimizations (colored yellow). The yellow regions show the windows of operability where the components can be set to meet the requirements for both responses (particle size and percent yield (Figure 10) for poly-l-lactide-based nanoparticles and particle size and surface zeta potential (Figure 11) for poly-ɛ-caprolactone-based nanoparticles).

## 5. Conclusions

One mission of a drug product development scientist is to develop drug delivery systems that enhance the optimal performance of bioactive agents. Many strategies are used to accomplish this purpose, including measuring the effect of several combinations of formulation and process variables on the properties of nanoparticles. By carefully selecting which combinations of these variables to evaluate, it is possible to optimize nanoparticle properties for specific purposes as embodied in quality by design (QbD) and process analytical technology (PAT) in pharmaceutical dosage form design and development. We have used d-optimal mixture statistical experimental design of experiments and analyze data in two types of nanoparticles (poly-l-lactide-based nanoparticles and poly-ɛ-caprolactone-based nanoparticles) in which the components are in proportions. The negative terms in the empirical model (Equation (1)) corresponding to the amounts of crosslinking agent and stabilizer included in the reaction mixture are the terms to be controlled for particle size minimization. Further, the resulting model (Scheffe polynomial) shown in Equation (2) indicates that terms with positive sign (initiator and stabilizer) will increase the yield if increased. The same reasoning is true in other models: Equations (3) and (4).

Following simultaneous numerical and graphical optimizations of the two models generated (Scheffe polynomials), the optimum formulations were identified.
